# Prevalence and risk factors of recurrent respiratory tract infections in preschool children: a systematic review and meta-analysis

**DOI:** 10.1016/j.jped.2026.101598

**Published:** 2026-09-05

**Authors:** Haizhu Huang, Xiaozhan Chen, Yi Wang, Dongxu Wang, Chuanlin Zhou

**Affiliations:** a The First People's Hospital of Yulin, Department of Respiratory and Critical Care Medicine, Yulin, Guangxi, China; b The First People's Hospital of Yulin, Department of Endoscopy, Yulin, Guangxi, China

**Keywords:** Preschool children, Recurrent respiratory tract infections, Risk factors, Meta-analysis, Systematic review

## Abstract

**Objective:**

To evaluate the prevalence and risk factors for recurrent respiratory tract infections in preschool children.

**Methods:**

Studies on RRTIs in preschool children were retrieved from PubMed, Cochrane Library, Embase, Web of Science, CBM, CNKI, Wanfang, and VIP databases up to October 2025. Data was analyzed using Stata 15.0.

**Results:**

Ten studies involving 15,271 preschoolers were included. Meta-analysis showed that the overall prevalence was 24.14% [95% CI (0.23,0.33)].A total of 11 risk factors for RRTIs in preschool children were identified, including: asthma (I² = 93.8%) [OR = 3.84, 95% CI (1.97, 7.47), *P* < 0.001], allergy(I² = 0) [OR = 2.27, 95% CI (2.06, 2.51), *P* < 0.001], dietary bias (I² = 65%) [OR = 2.10, 95% CI (1.37, 3.24), *P* = 0.001], smoking co-resident(I² = 42.7%) [OR = 1.83, 95% CI (1.44, 2.32), *P* < 0.001], low parental education level(I² = 0) [OR = 3.17, 95% CI (1.76, 5.69), *P* < 0.001], initial use of antibiotics < 6 months(I² = 0) [OR = 1.70, 95% CI (1.50, 1.93), *P* < 0.001], maternal BMI per 3 kg/m² increment(I² = 0) [OR = 1.18, 95% CI (1.08, 1.28), *P* < 0.001], and breastfeeding duration < 6 months(I² = 0) [OR = 1.26, 95% CI (1.12, 1.42), *P* < 0.001].

**Conclusion:**

The high prevalence (24.14%) of RRTIs in preschool children and the risk factors identified provide evidence for targeted prevention and intervention strategies.

## Introduction

Respiratory tract infections (RTIs) have consistently posed a serious threat to children's health and represent a leading cause of mortality, constituting a major burden in pediatric outpatient and inpatient settings, particularly in developing countries [1]. RTIs encompass upper respiratory tract infections (URTIs) and lower respiratory tract infections (LRTIs). These infections significantly reduce the quality of life of affected children and their families, while imposing substantial personal, medical, and economic burdens on patients, their caregivers, and the healthcare system [2]. RTIs cause discomfort and pain in affected children and may impede their growth and development [3]; Severe cases can lead to hospitalization or even death [4]; Recurrent infections further increase the risk of complications, including antibiotic overuse and antimicrobial resistance [5]; and may also contribute to conditions such as edema, cough, and asthma [6]. When RTIs occur, parents often need to devote substantial time to caring for their sick children, resulting in work absenteeism [5]; Moreover, long-term caregiving and persistent concerns regarding their child’s health impose considerable psychological stress on the family [7]. Frequent outpatient visits and hospitalizations impose a substantial economic burden; when treatment proves ineffective and complications arise, the strain on the healthcare system is further exacerbated [6]. Preschool children are generally susceptible to respiratory infections due to immune immaturity and the underdevelopment of their respiratory systems, and most infections are viral and self-limiting in nature, it is estimated that up to 25% of children experience recurrent respiratory tract infections (RRTIs) within their first four years of life, which is considered a common developmental phenomenon [3]. In routine pediatric practice, no underlying pathology could be identified in 64% of 208 children presenting with RRTIs, the most frequently observed coexisting conditions were asthma/preschool wheezing or adenoid hypertrophy, while severe primary immunodeficiencies or pulmonary diseases were rarely identified. With advancing age and progressive maturation of the immune system, symptoms generally resolve spontaneously in the majority of cases [8]. But RRTIs are common in early childhood and represent one of the most prevalent diseases affecting preschool children (aged <7 years) [2]. It is estimated that 10–15% of all children experience RRTIs [9], with recurrent lower respiratory tract infections (e.g., pneumonia) constituting a leading infectious cause of mortality in children under five years of age [2]. This contributes considerably to the global burden of infectious diseases and exerts a profound impact on preschool-aged children, accounting for an estimated 704,000 deaths annually in this age group and resulting in a loss of 6.06 billion disability-adjusted life years (DALYs) [10]. RRTIs are also highly prevalent in otherwise healthy children. In developed countries, up to 25% of children under one year of age and approximately 18% of children aged 1–4years experience RRTIs [2]. The typical incidence ranges from 3 to 8 episodes of URTIs per year, with approximately 10–15% of children experiencing at least 12 infections annually [11]. Currently, no universal diagnostic criteria exist for RRTIs in children. The definitions of RRTIs vary across different studies and guidelines, as detailed below: (1) The 1990 Graham criteria: [12] These criteria do not distinguish between upper and lower respiratory tract infections, tracheobronchitis, or pneumonia, but instead set specific frequency thresholds for localized infections: otitis media is defined as ≥ 3 episodes/month or ≥ 4/year, infectious rhinitis as > 5/year, and pharyngitis/tonsillitis as > 3/year. (2) The 1993 De Mattia et al. criteria: [13] This definition sets overall RTIs at ≥ 6/year, upper RTIs at ≥ 1/month (September–April), recurrent tracheobronchitis at ≥ 3/year, and recurrent pneumonia at ≥ 3/year, without addressing otitis media, rhinitis, pharyngitis, or tonsillitis. (3) The 2008 criteria proposed by the Respiratory Group of the Pediatric Society of the Chinese Medical Association provide age-stratified thresholds for upper respiratory infections (7/year at 0–2 years, 6/year at 2–5 years, 5/year at 5–14 years), recurrent tracheobronchitis (3/year at 0–2 years, 2/year thereafter), and recurrent pneumonia (2/year for all ages), but do not cover otitis media, rhinitis, pharyngitis, or tonsillitis [14]. In this systematic review, we used the 2008 criteria proposed by the Respiratory Group of the Pediatric Society of the Chinese Medical Association as the core definition of RRTIs. Therefore, it is essential to thoroughly investigate the current status and risk factors of RRTIs in preschool children. Distinguishing physiological respiratory morbidity from true RRTIs is critical to preventing overdiagnosis, overtreatment, and unnecessary antibiotic use. Such investigation not only facilitates the formulation of individualized treatment strategies but also contributes to the effective prevention of complications, improves the quality of life for affected children and their families, and reduces the societal healthcare burden. Although numerous studies have examined the risk factors for RRTIs in preschool children, the reported risk factors vary considerably across studies. This study employs a meta-analytic approach to assess the prevalence and risk factors of RRTIs in this population, with the aim of providing robust evidence to inform the development of targeted intervention strategies.

## Materials and methods

This protocol has been registered in the International Prospective Register of Systematic Reviews (PROSPERO: CRD420251176577).

### Search strategy

A computerized literature search was conducted across the following databases: PubMed, Cochrane Library, Embase, Web of Science, China Biology Medicine (CBM), China National Knowledge Infrastructure (CNKI), Wanfang Data Knowledge Service Platform, and VIP Chinese Journal Service Platform. The search aimed to identify published studies on the prevalence and risk factors of recurrent respiratory tract infections in preschool children, with the timeframe covering from database inception to October 2025. The search strategy employed a combination of subject headings and free-text keywords. The Chinese search terms were “学龄前儿童” and “反复呼吸道感染”. Detailed search strategies for Embase, Web of Science, and PubMed are provided in Supplementary Tables S1, S2, and S3, respectively.

### Inclusion and exclusion criteria

Inclusion criteria: (1) the study population comprised preschool children; (2) the study objective was to investigate risk factors for recurrent respiratory tract infections; (3) the study design was an observational study (case–control study); (4) the study outcome measures included at least one risk factor or predictor for recurrent respiratory tract infections in preschool children. Exclusion criteria: (1) duplicate publications; (2) the study type was Meta-analysis, review, case report, irrelevant research; (3) study for which did not report the outcomes of interest; (4) studies of low methodological quality; (5) study that the full text was not available.

### Data extraction

Two reviewers independently conducted literature screening and data extraction. Initial screening was performed by reading titles and abstracts, followed by full-text review for further assessment. The extracted data were cross-checked between the two reviewers; any disagreements were resolved by consultation with a third reviewer. Literature selection was strictly conducted in accordance with the predefined inclusion and exclusion criteria. Data extracted from each eligible study included: first author, year of publication, country, study design, study population, number of included cases, sex distribution, and risk factors.

### Quality assessment

Two reviewers independently assessed the methodological quality of the included studies. The Newcastle-Ottawa Scale (NOS) [15] was employed to evaluate case–control studies, which assesses three domains: selection of study groups (4 points) and ascertainment of either the exposure or outcome (3 points). The total possible score is 9 points, with scores ≤ 4, 5–6, and ≥ 7 indicating low, moderate, and high quality, respectively. In the event of disagreements between the two reviewers regarding the assessment process, consensus was reached through discussion or by consulting a third reviewer. Only studies of moderate or high quality were included in the analysis.

### Statistical analysis

Statistical analysis was performed using Stata version 15.0 software. Odds ratios (ORs) with 95% confidence intervals (CIs) were used as effect measures for dichotomous outcomes. Statistical heterogeneity among the included studies was assessed using the Q test and I² statistic. If *P* ≥ 0.1 and I² ≤ 50%, indicating no substantial statistical heterogeneity, a fixed-effects model was applied for meta-analysis. If P < 0.1 or I² > 50%, indicating significant statistical heterogeneity, a random-effects model was employed. Publication bias was evaluated using funnel plots, and sensitivity analysis was conducted by sequentially omitting individual studies.

## Results

### Literature search results

**Figure 1:**
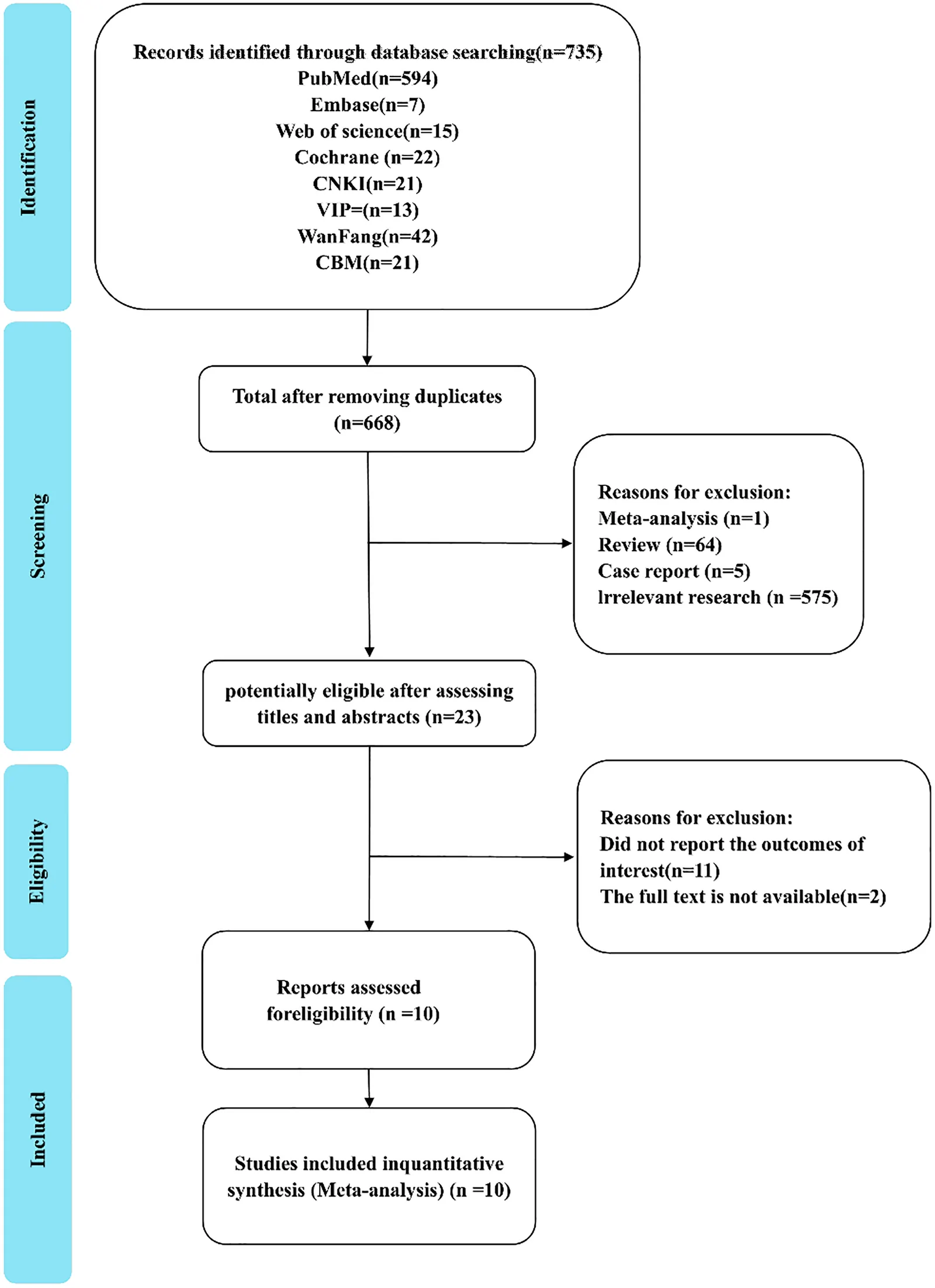
Flow chart of the literature search.

A total of 735 records were initially identified. After removing 67 duplicate records, 668 records were screened based on titles and abstracts, resulting in the exclusion of 645 records. Following full-text review of the remaining 23 records, 13 articles were excluded. Ultimately, 10 studies [16, 17, 18, 19, 20, 21, 22, 23, 24, 25] were included in the meta-analysis. The literature selection process is illustrated in Figure 1.

### Characteristics and quality assessment of the included studies

All 10 included studies [16, 17, 18, 19, 20, 21, 22, 23, 24, 25] were case–control studies, comprising a total of 15,271 preschool children, with all studies originating from China. Among these participants, there were 3687 patients with RRTIs. A total of 11 risk factors for recurrent respiratory tract infections in this population were identified. The results of the quality assessment showed that the NOS scores ranged from 7 to 8(Supplementary Tables S4 provides the detailed scoring table for each included study). The basic characteristics and quality assessment scores of the included studies are presented in Table 1.

**Table 1:** Basic features and literature quality scores of the involved literature.

Author	Year	Country	Study design	Sample size		Gender (Male/Female)		Risk factor	NOS
				RRTIs	NO-RRTIs	RRTIs	NO-RRTIs		
Zhou B [16]	2020	China	case control	1727	5495	836/891	2765/2730	①②⑨⑩⑪	8
Jin HX [17]	2012	China	case control	180	540	105/75	315/225	①③④⑤	7
Liu pH [18]	2017	China	case control	432	1151	258/174	654/497	①④⑤⑦	8
Chen YN [19]	2019	China	case control	168	696	94/74	387/309	③⑤⑧	8
Qi F [20]	2020	China	case control	137	137	78/56	75/62	①②⑥	8
Tao SZ [21]	2020	China	case control	71	75	38/33	39/36	④⑤⑦	7
Li XY [22]	2021	China	case control	240	1039	64/57	596/572	③⑥	8
Zhou LM [23]	2021	China	case control	522	1686	290/232	977/709	①②⑨⑩⑪	8
He J [24]	2022	China	case control	137	138	64/73	67/71	③⑤ ⑥	7
Li SF [25]	2016	China	case control	73	627	-	-	③④⑤⑧	7

Tips: RRTIs, Recurrent Respiratory Tract Infections, NO-RRTIs, No Recurrent Respiratory Tract Infections, NOS, Newcastle–Ottawa Scale. The following factors were included in the table: ①Asthma, ②Allergy, ③Dietary bias, ④Co-resident with chronic respiratory disease,⑤Smoking co-resident,⑥Preterm birth, ⑦Daily outdoor activity time ≤ 2 h,⑧Low parental education level, ⑨Initial use of antibiotic <6 months, ⑩Maternal BMI per 3 kg/m2 increment, ⑪Breast feeding duration <6 months.

### Prevalence of RRTIs in preschool children

**Figure 2:**
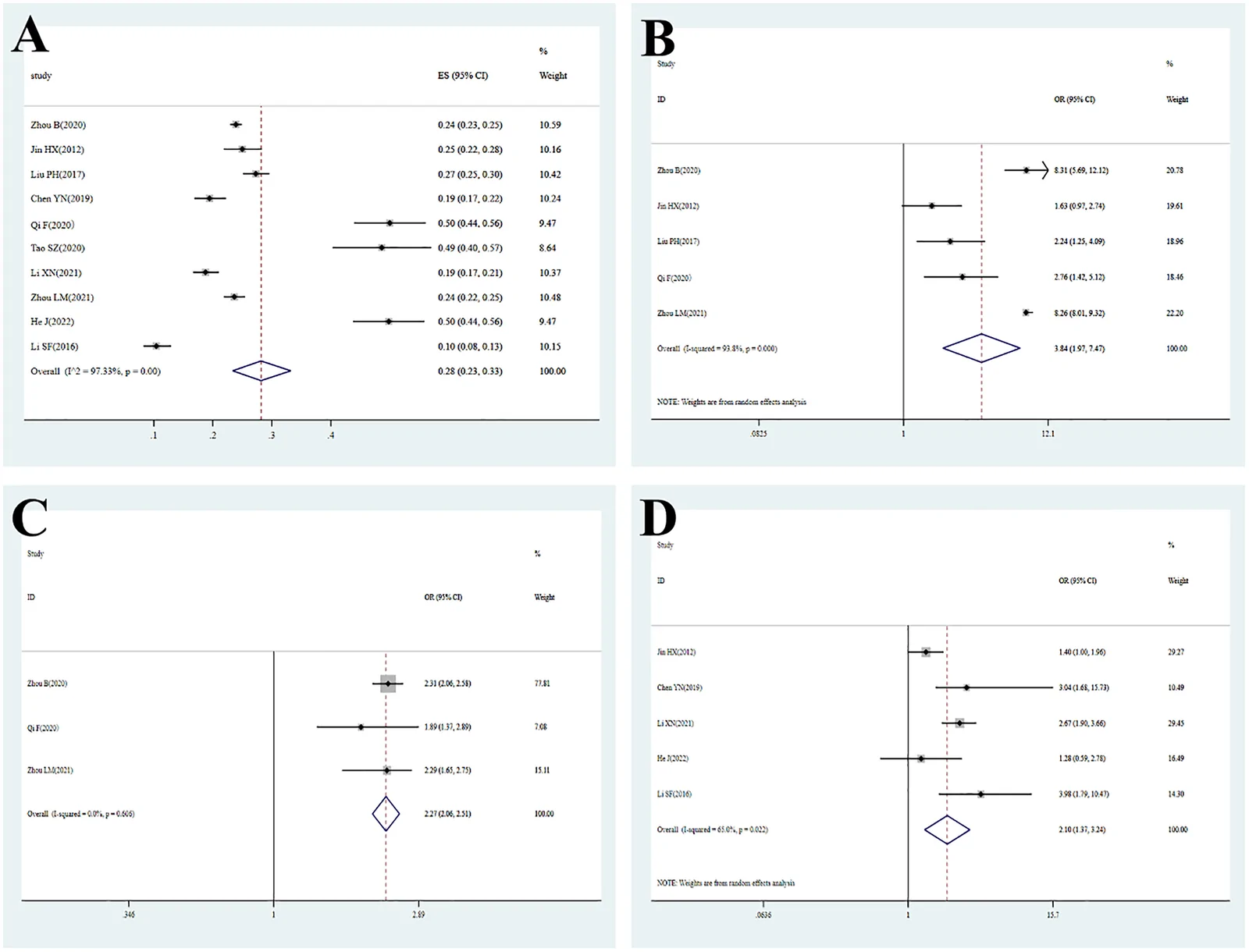
Forest plots of meta-analyses for prevalence and risk factors. (A) Prevalence: Forest plot of ten studies. The overall pooled ES is 0.28 (95% CI: 0.23–0.33), indicating significant heterogeneity (I² = 97.33%, p<0.01). (B) Asthma: Forest plot of five studies. The overall pooled OR is 3.84 (95% CI: 1.97–7.47), with substantial heterogeneity (I² = 93.8%, p<0.001). (C) Allergy: Forest plot of three studies. The overall pooled OR is 2.27 (95% CI: 2.06**–**2.51), with no significant heterogeneity (I² = 0, *P* = 0.606). (D) Dietary bias: Forest plot of five studies. The overall pooled OR is 2.10 (95% CI: 1.37–3.24), with moderate heterogeneity (I² = 65.0%, *P* = 0.022).

All 10 included studies [16, 17, 18, 19, 20, 21, 22, 23, 24, 25] reported the prevalence of RRTIs in preschool children. Significant statistical heterogeneity was observed among the studies (I² = 97.33%, *P* < 0.01); therefore, a random-effects model was employed for meta-analysis. The results showed that the pooled prevalence of RRTIs in preschool children was 24.14% [95% CI (0.23,0.33)] (Table 2; Figure 2A).

**Table 2:** Detailed data on 11 inclusion factors for RRTIs in preschool children.

Inclusion factors	Literatures (n)	Heterogeneity test		Effect model	OR (95%CI)	P-value	Reference	
		I^2^%	P-value					
Asthma	5	93.8	<0.001	Random effects	3.84(1.97,7.47)	<0.001	[16, 17, 18,20,23]	
Allergy	3	0	0.606	Fixed effects	2.27(2.06,2.51)	<0.001	[16,20,23]	
Dietary bias	5	65	0.022	Random effects	2.10(1.37,3.24)	0.001	[17,19,22,24,25]	
Co-resident with chronic respiratory disease	4	68.3	0.024	Random effects	1.57(0.87,2.81)	0.132	[17,18,21,25]	
Smoking co-resident	6	42.7	0.121	Fixed effects	1.83(1.44,2.32)	<0.001	[17, 18, 19,21,24,25]	
Preterm birth​	3	83.5	0.002	Random effects	1.14(0.37,3.52)	0.816	[20,22,24]	
Daily outdoor activity time ≤ 2 h	2	85.1	0.009	Random effects	1.29(0.53,3.17)	0.574	[18,21]	
Low parental education level	2	0	0.629	Fixed effects	3.17(1.76,5.69)	<0.001	[19,25]	
Initial use of antibiotic <6 months	2	0	0.78	Fixed effects	1.70(1.50,1.93)	<0.001	[16,23]	
Maternal BMI per 3 kg/m2 increment	2	0	0.661	Fixed effects	1.18(1.08,1.28)	<0.001	[16,23]	
Breast feeding duration <6 months	2	0	0.728	Fixed effects	1.26(1.12,1.42)	<0.001	[16,23]	

Tips: *P* < 0.05, indicating that this factor is a risk factor for RRTIs.

### Meta-analysis of risk factors for RRTIs in preschool children

#### Asthma

Five studies [16, 17, 18, 20, 23] reported asthma as a risk factor for RRTIs in preschool children, and the heterogeneity test (I² = 93.80%, *P* < 0.001) was performed using a random-effects model. The results indicated that asthma was a significant risk factor for RRTIs in preschool children [OR = 3.84, 95% CI (1.97, 7.47), *P* < 0.001] (Figure 2B).

#### Allergy

Three studies [16,20,23] identified allergy as a risk factor for RRTIs in preschool children, and the heterogeneity test (I² = 0, *P* = 0.606) was performed based on the fixed-effects model. It was found that allergy was a risk factor for RRTIs in preschool children, and there exists an obvious difference [OR = 2.27, 95% CI (2.06, 2.51), *P* < 0.001] (Figure 2C).

#### Dietary bias

Five studies [17, 19, 22, 24, 25] mentioned dietary bias as a risk factor for RRTIs in preschool children, and the heterogeneity test (I² = 65.0%, *P* = 0.022) was performed based on the random-effects model. The results of the analysis reflected that dietary bias was a significant risk factor for RRTIs in preschool children [OR = 2.10, 95% CI (1.37, 3.24), *P* = 0.001] (Figure 2D).

#### Co-resident with chronic respiratory disease

**Figure 3:**
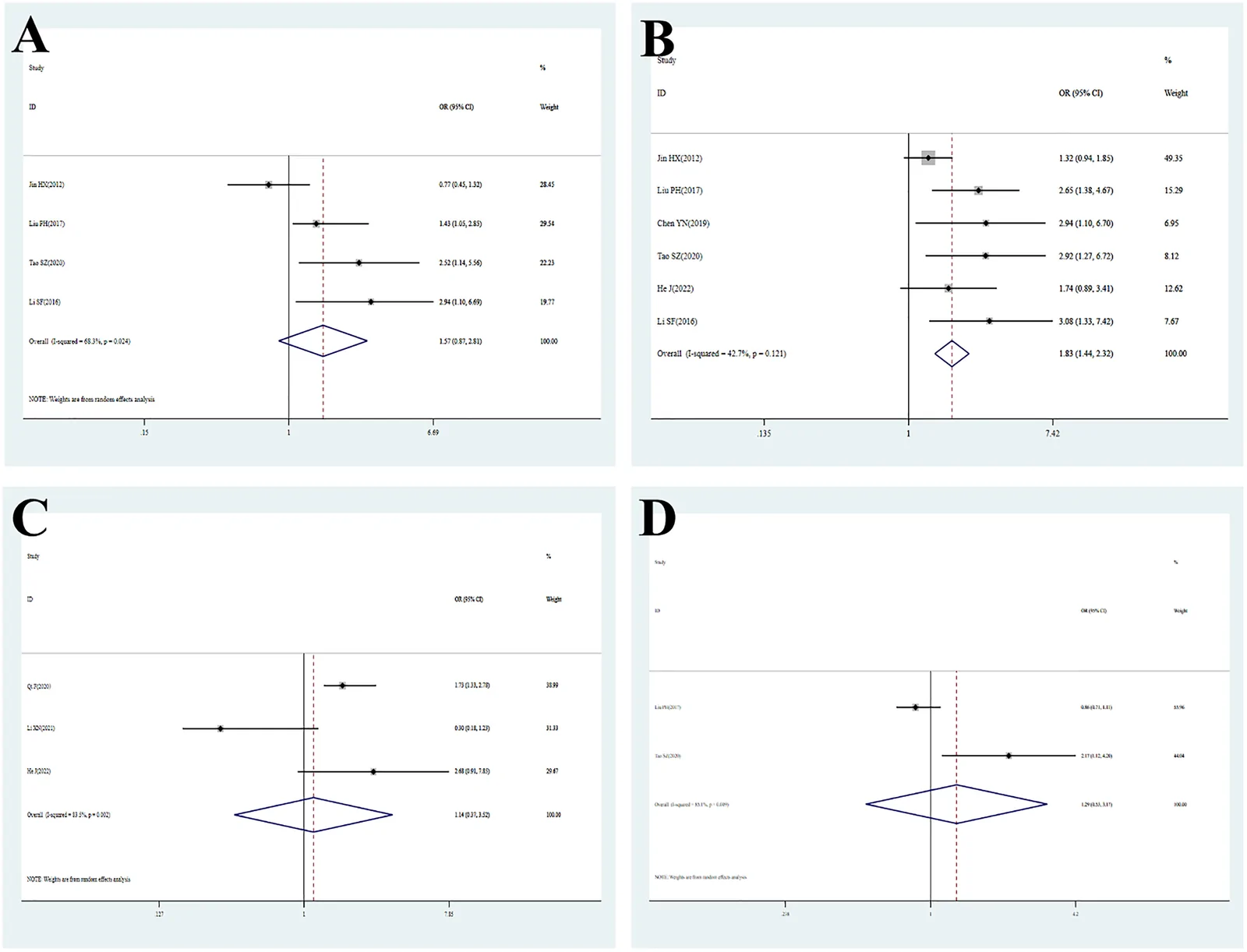
Forest plots of meta-analyses for environmental and lifestyle factors. (A) Co-resident with chronic respiratory disease: Forest plot of 4 studies. The overall pooled OR is 1.57 (95% CI: 0.87–2.81), indicating moderate heterogeneity (I² = 68.3%, *P* = 0.024). (B) Smoking co-resident: Forest plot of 6 studies. The overall pooled OR is 1.83 (95% CI: 1.44–2.32), with low heterogeneity (I² = 42.7%, *P* = 0.121). (C) Preterm birth: Forest plot of 3 studies. The overall pooled OR is 1.14 (95% CI: 0.37–3.52), with significant heterogeneity (I² = 83.5%, *P* = 0.002). (D) Daily outdoor activity time ≤ 2 h: Forest plot of 2 studies. The overall pooled OR is 1.29 (95% CI: 0.53–3.17), with moderate heterogeneity (I² = 85.1%, *P* = 0.0009).

Four studies [17,18,21,25] investigated co-residence with individuals having chronic respiratory disease as a risk factor for RRTIs in preschool children, and the heterogeneity test (I² = 68.3%, *P* = 0.024) was conducted based on the random-effects model. The results suggested that co-residence with individuals having chronic respiratory disease was not a significant risk factor for RRTIs in preschool children [OR = 1.57, 95% CI (0.87, 2.81), *P* = 0.132] (Figure 3A).

#### Smoking co-resident

Six studies [17, 18, 19,21,24,25] mentioned smoking co-resident as a risk factor for RRTIs in preschool children, and the heterogeneity test (I² = 42.7%, *P* = 0.121) was conducted based on the fixed-effects model. It was found that smoking co-resident was a risk factor for RRTIs in preschool children, with obvious difference [OR = 1.83, 95% CI (1.44, 2.32), *P* < 0.001] (Figure 3B).

#### Preterm birth

Preterm birth [20,22,24] was mentioned as a risk factor in three studies, and the heterogeneity test (I² = 83.5%, *P* = 0.002) was performed based on the random-effects model. The results indicated that preterm birth was not a statistically significant risk factor for RRTIs in preschool children [OR = 1.14, 95% CI (0.37, 3.52), *P* = 0.816] (Figure 3C).

#### Daily outdoor activity time ≤ 2 h

Two studies [18,21] investigated daily outdoor activity time ≤ 2 h as a risk factor for RRTIs in preschool children, and the heterogeneity test (I² = 85.1%, *P* = 0.009) was conducted based on the random-effects model. It was found that daily outdoor activity time ≤ 2 h was not a significant risk factor for RRTIs in preschool children [OR = 1.29, 95% CI (0.53, 3.17), *P* = 0.574] (Figure 3D).

#### Low parental education level

**Figure 4:**
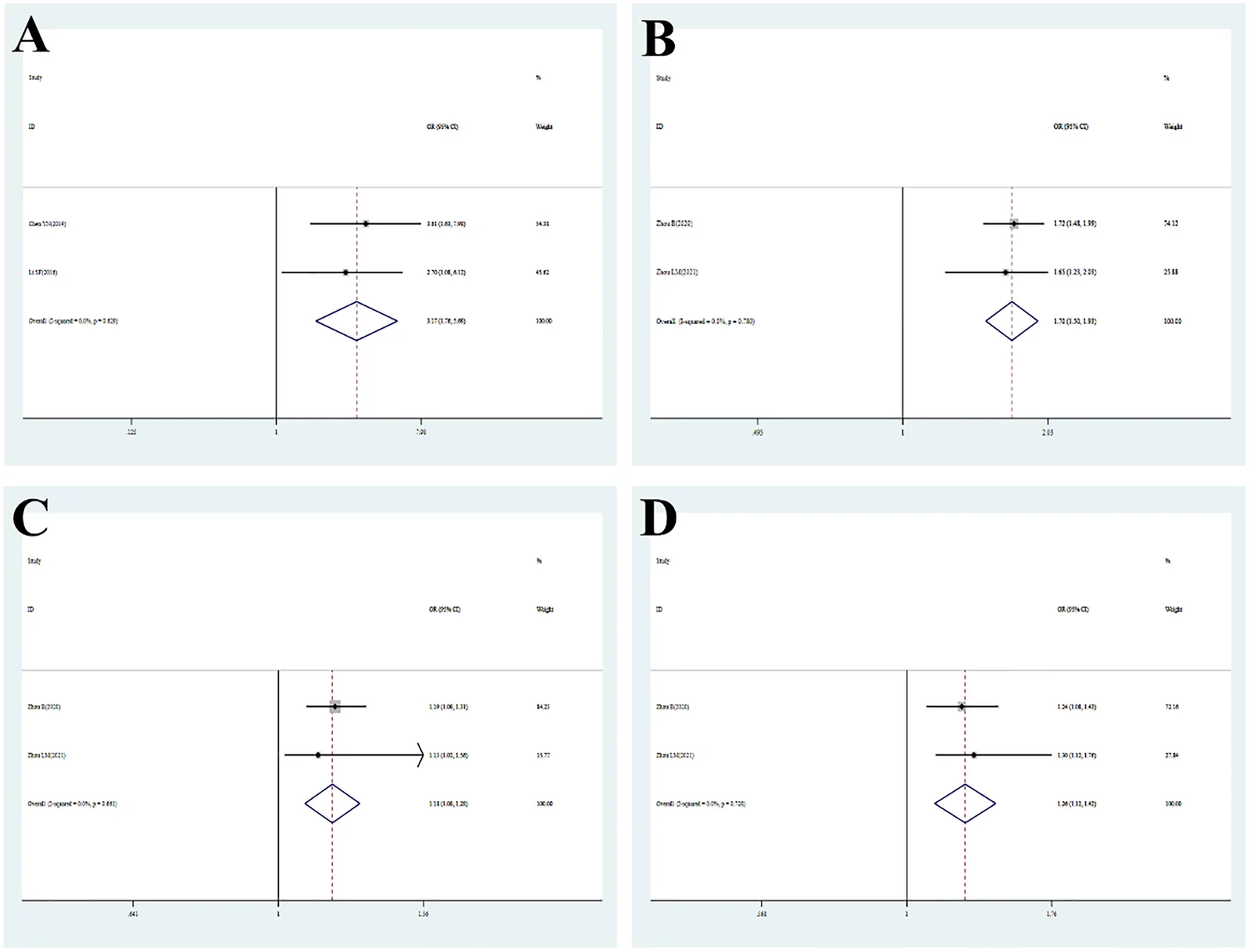
Forest plots of meta-analyses for early-life and parental factors. (A) low Parental education level: Forest plot of two studies. The overall pooled OR is 3.17 (95% CI: 1.76–5.69), with no significant heterogeneity (I² = 0, *P* = 0.629). (B) Initial use of antibiotic <6 months: Forest plot of two studies. The overall pooled OR is 1.70 (95% CI: 1.50–1.93), with no significant heterogeneity (I² = 0, *P* = 0.780). (C) Maternal BMI per 3 kg/m² increment: Forest plot of two studies. The overall pooled OR is 1.18 (95% CI: 1.08–1.28), with no significant heterogeneity (I² = 0, *P* = 0.661). (D) Breast feeding duration <6 months: Forest plot of two studies. The overall pooled OR is 1.26 (95% CI: 1.12–1.42), with no significant heterogeneity (I² = 0, *P* = 0.728).

Two studies [19,25] mentioned low parental education level as a risk factor for RRTIs in preschool children, and the heterogeneity test (I² = 0, *P* = 0.629) was conducted based on the fixed-effects model. The results reflected that low parental education level was a significant risk factor for RRTIs in preschool children [OR = 3.17, 95% CI (1.76, 5.69), *P* < 0.001] (Figure 4A).

#### Initial use of antibiotic < 6 months

Two studies [16,23] reported Initial use of antibiotic < 6 months as a risk factor for RRTIs in preschool children, and the heterogeneity test (I² = 0, *P* = 0.78) was performed based on the fixed-effects model. The results suggested that Initial use of antibiotic < 6 months was a statistically significant risk factor for RRTIs in preschool children [OR = 1.70, 95% CI (1.50, 1.93), *P* < 0.001] (Figure 4B).

#### Maternal BMI per 3 kg/m² increment

Two studies [16,23] identified maternal BMI per 3 kg/m² increment as a risk factor for RRTIs in preschool children, and the heterogeneity test (I² = 0, *P* = 0.661) was conducted based on the fixed-effects model. It was found that maternal BMI per 3 kg/m² increment was a risk factor for RRTIs in preschool children, and there exists an obvious difference [OR = 1.18, 95% CI (1.08, 1.28), *P* < 0.001] (Figure 4C).

#### Breast feeding duration <6 months

Two studies [16,23] mentioned breast feeding duration < 6 months as a risk factor for RRTIs in preschool children, and the heterogeneity test (I² = 0, *P* = 0.728) was performed based on the fixed-effects model. The results of the analysis indicated that breast feeding duration < 6 months was a risk factor for RRTIs in preschool children, with obvious difference [OR = 1.26, 95% CI (1.12, 1.42), *P* < 0.001] (Figure 4D).

### Publication bias and sensitivity analysis

Funnel plots and sensitivity analyses were shown for (A) prevalence, (B) asthma, (C) dietary bias, (D) co-resident with chronic respiratory disease, (E) preterm birth, and (F) daily outdoor activity time ≤ 2 h. The symmetrical distribution of points within the pseudo 95% confidence limits (dashed lines) indicated a low risk of publication bias and supports the robustness of the meta-analysis results for all examined associations (Supplementary Figure S1). Each plot displays the pooled estimate (dot) and 95% confidence interval (horizontal line) when individual studies were sequentially omitted. The stability of the overall estimates across all analyses confirms the robustness of the meta-analysis results (Supplementary Figure S2).

## Discussion

### Prevalence of RRTIs in preschool children

RRTIs represent a common clinical condition in pediatric populations. According to the literature [3], approximately 25% of children experience RRTIs within the first four years of life. Another study [26] reported that RRTIs occur in about 25% of infants under one year of age and 6% of children prior to age six. Most affected children present with mild clinical symptoms, and the frequency of episodes tends to decrease progressively with age, with complete resolution typically observed by the age of 12 years. In summary, the prevalence of RRTIs in preschool children varies across age groups and populations, but remains at a relatively high level overall (approximately 6%–25%). The findings of the present study indicate that the pooled prevalence of RRTIs in preschool children is 24.14%, which is generally consistent with the upper range reported in the literature, further confirming the high incidence of RRTIs in this population. Consequently, the high prevalence of RRTIs among preschool children underscores the urgent need to develop and implement effective preventive and therapeutic interventions. Addressing RRTIs in the pediatric population is essential to mitigating their substantial health, economic, and social repercussions.

### Risk factors for RRTIs in preschool children

#### Biological susceptibility factors

The findings of this study indicate that asthma [OR = 3.84] and allergy [OR = 2.27] are risk factors for RRTIs in preschool children. The immune system in children is inherently predisposed to recurrent and persistent infections [27,28]. Allergic diseases, such as asthma, increase susceptibility to respiratory tract infections through multiple interrelated mechanisms. First, the airway epithelial barrier is typically impaired in individuals with allergic diseases [29]. Specifically, the airway epithelium constitutes a physical barrier that prevents the invasion of pathogens and allergens. In patients with asthma, chronic airway inflammation and structural remodeling are frequently observed. This chronic inflammation and structural remodeling —including goblet cell hyperplasia and ciliary damage — further compromise barrier function, manifesting as epithelial barrier dysfunction and impaired ciliary activity. These alterations facilitate pathogen penetration across the epithelial layer, thereby weakening the physical defense mechanisms of the respiratory tract against pathogens [30, 31, 32]. Second, the immune system in these patients tends to favor a Th2-dominant response, characterized by excessive production of IL-4, IL-5, and IgE, leading to enhanced eosinophilic infiltration. This Th2-skewed response diminishes the efficacy of Th1-mediated immune defense against viral and bacterial pathogens [33]. Furthermore, patients with asthma often require corticosteroids to manage symptoms. Although corticosteroids are effective in suppressing local airway inflammation, their long-term use can suppress both local and systemic immune function [34]. In addition, in children — particularly those under one year of age — acquired immunity is not yet fully developed, resulting in relatively low immune competence. Collectively, the combined effects of impaired barrier function and immune disbalance significantly increase susceptibility to respiratory tract infections in individuals with allergic diseases.

#### Early-Life exposure factors

The findings of this study indicate that initial use of antibiotics < 6 months [OR = 1.70], maternal BMI per 3 kg/m² increment [OR = 1.18], and breastfeeding duration < 6 months [OR = 1.26] are risk factors for RRTIs in preschool children. Although antibiotics confer significant health benefits in the treatment or prevention of bacterial infections, accumulating evidence suggests that they exert disruptive effects on host–microbiota homeostasis, posing a serious threat to global public health [35]. During infancy, due to the immaturity of the microbiota and the incomplete functional development of the immune system, the consequences of antibiotic treatment are more severe and persistent than those observed in adults [27]. This is primarily manifested in the following aspects: (1) Disruptive effects on the gut microbiota. The infant gut microbiota remains in a state of instability during the first 2–3 years of life [35]. Early-life antibiotic exposure disrupts the typical maturation trajectory of the gut microbiota, increases the abundance of antibiotic-resistant bacteria, enriches the resistance gene reservoir within the gut microbial community, and exerts adverse effects on child health [36]. A prospective controlled cohort study demonstrated that the timing of antibiotic exposure appears to be a critical determinant of gut microbiota alterations, with perinatal antibiotic exposure exerting a more pronounced impact on the gut microbiota of one-year-old infants than exposure occurring later in life [37]. (2) Long-term effects on immune system development. Antibiotic exposure during infancy may induce lasting consequences through microbiota–immune system crosstalk, potentially depleting key microbial taxa critical for normal immune maturation and leading to impaired local and systemic immune competence [38], Such disruption may affect both innate and adaptive immune responses to pathogens in infants [35], and has been associated with an increased risk of allergic diseases in childhood, including atopic dermatitis and asthma [39,40]. An additional cohort study confirmed that, compared with controls, children with early-life antibiotic exposure exhibited higher rates of subsequent infections and antibiotic usage later in childhood [41]. Although antibiotics are critical for infection treatment, their long-term effects on microbiome and immune development must be weighed. We therefore recommend a conservative antibiotic approach in infants and young children, and highlight the need to balance the risks and benefits of neonatal antibiotic exposure.

Due to the increasing prevalence of obesity and the trend of delayed childbearing, the number of children born to overweight or obese mothers is steadily rising [42,43]. Maternal obesity influences offspring susceptibility to long-term pediatric infectious morbidity [44]. Accumulating evidence suggests that children of obese mothers are at higher risk of hospitalization for respiratory diseases [45], including asthma [46] and respiratory symptoms such as wheezing [47]. The UK Bradford birth cohort study showed that higher maternal BMI was associated with an increased risk of respiratory tract infections (including lower respiratory tract infections, asthma, and otitis media) in offspring within the first year of life, with greater risk corresponding to higher obesity grades (class I obesity: RR = 1.18; class II obesity: RR = 1.31) [48]. A large Danish cohort study (n = 688,457) found that maternal BMI during early pregnancy was a risk factor for lower respiratory tract infections in offspring, particularly among children with an allergic predisposition [49]. Maternal pregnancy obesity has been associated with an increased risk of recurrent lower respiratory tract infections (e.g., bronchitis, pneumonia) in offspring during infancy, with a more pronounced effect observed among minority populations in low-income urban areas [50]. The potential underlying mechanisms may include the following. Studies have confirmed that offspring of obese mothers exhibit reduced pulmonary insulin sensitivity and elevated levels of allergen-specific IgE and IL-5 [51]. Furthermore, maternal BMI may influence fetal immune development through inflammatory states, metabolic abnormalities, or placental function, thereby increasing susceptibility to infections [52]. Maternal obesity during pregnancy leads to metabolic syndrome, chronic inflammation, and endocrine alterations, which can modify placental function and the maternal gut microbiome, as well as induce inflammation in the placenta and intrauterine environment. All of these factors collectively influence fetal growth and development, including brain and lung development [53]. From a population perspective, the finding suggests that maternal pre-pregnancy obesity may have a certain impact on children's respiratory health, warranting attention in public health research. However, its small effect size (OR = 1.18) limits its clinical utility, and it should not be a pediatric priority. Weight management in women of childbearing age should be pursued through preconception and population health efforts, rather than as a primary target in RRTI care.

A Nigerian study demonstrated that, compared with children breastfed for 1–6 months, those breastfed for 19–24 months exhibited significantly lower odds of recent acute respiratory illnesses after breastfeeding cessation (AOR = 0.37, 95% CI [0.15–0.79], *P* = 0.04). However, no significant difference was observed between feeding for 1–6 months and other shorter durations (e.g., 7–12 months) (*P* > 0.05) [54], suggesting that breastfeeding for <6 months may be insufficient to confer long-term immune protection. A prospective case–control study from China showed that among infants under 6 months of age, longer duration of exclusive breastfeeding was associated with lower recurrence rates of pneumonia, shorter hospital stays, and reduced hospitalization costs [55]. Early postnatal nutrition — particularly lactoferrin in breast milk — has profound implications for both development and long-term health, playing a crucial role in the growth of various biological systems and the prevention of numerous chronic diseases. Insufficient breastfeeding duration leads to lactoferrin deficiency, which may subsequently result in nutritional inadequacies and adversely affect the gut microbiota and immune pathways [56]. SIgA is the most abundant immunoglobulin subtype on mucosal surfaces and plays an important role in defending against pathogen invasion. Insufficient breastfeeding leads to reduced levels of SIgA in the infant respiratory and intestinal tracts, compromising the first-line mucosal defense and increasing the risk of respiratory mucosal immune disorders [57]. Breastfeeding is a key determinant in shaping the infant gut microbiota, and inadequate breastfeeding duration is associated with reduced microbial diversity and an increased abundance of potential pathogens (e.g., Enterobacteriaceae) [58]. In conclusion, breastfeeding reduces the risk of childhood respiratory diseases by modulating respiratory mucosal immunity and the gut microbiota while enhancing immune responses. Breastfeeding for less than six months may be insufficient to fully establish these protective mechanisms.

#### Family-Social environment and behavioral factors

The findings of this study indicate that smoking co-resident [OR = 1.83], low parental education level [OR = 3.17], and dietary bias [OR = 2.10] are risk factors for RRTIs in preschool children. Preschool children, due to their immature immune systems, are a high-risk population for RRTIs. Passive smoking resulting from living with a smoking co-resident has been identified as one of the significant environmental risk factors [3]. A study from Spain reported that household secondhand smoke exposure was associated with significantly increased incidence and hospitalization rates for childhood asthma, otitis media, and lower respiratory tract infections [59]. Research from Poland also indicated that exposure to smoking environments in children under three years of age led to a significant increase in respiratory tract infections, as well as a higher incidence of otitis media. In households with smokers, children are more susceptible to passive secondhand smoke absorption, thereby significantly increasing the likelihood of frequent hospitalizations or antibiotic use due to respiratory infections [60]. When children co-reside with smokers, the passive smoking household environment may promote nasal biofilm formation, subsequently increasing the risk of persistent sinusitis and other respiratory tract infections [61]. Co-resident smokers may alter the microbial communities in the lower respiratory tract of children through secondhand smoke exposure, thereby increasing susceptibility to infections [62]. The impact of smoking on children's health is multifaceted. Exposing preschool children to smoking environments affects not only their physical health but also their development and behavior. For preschool children, such exposure is inequitable, and it is the responsibility of every parent to protect them from the harms of tobacco smoke.

A Finnish study investigating the relationship between parental education, income, employment status, and the risk of hospitalization for RSV bronchiolitis in infants demonstrated that lower parental education level significantly increased the risk of infant RSV hospitalization, with the risk increasing as education level decreased [63]. A study from Ethiopia showed that maternal occupation and education level were significantly associated with acute respiratory infections in children [64]. Parental knowledge regarding the management of respiratory tract infections may influence disease outcomes. Another study found that parents of children with frequent upper respiratory tract infections demonstrated a higher level of knowledge regarding wheezing management. During wheezing episodes, 91.8% of parents administered pharmacological treatment, while 80.1% used herbal remedies, suggesting that enhancing parental education may contribute to improved symptom recognition and management [65]. An association exists between parental education and childhood infectious diseases, with research indicating that parents with higher education levels are generally associated with higher coverage rates of voluntary vaccinations (e.g., varicella, mumps, and influenza) [66].

Dietary bias refers to an unreasonable and unhealthy dietary tendency in children, characterized by imbalanced dietary structure, uneven nutrient intake, or poor eating behaviors. Dietary bias can lead to deficiencies in immune-related nutrients such as vitamin A, vitamin D, calcium, and zinc, which may subsequently increase the risk of RRTIs through various immune mechanisms. A study from China demonstrated that low serum vitamin A concentration in children is associated with a high incidence of RRTIs; both low serum vitamin A concentration and current RTI symptoms are associated with the occurrence of RRTIs; and low intake of vitamin A-rich foods is also linked to the development of RRTIs [67]. It has been reported that vitamin A influences respiratory health by maintaining epithelial integrity and exerting anti-infective effects [68]. Additionally, studies have shown that low vitamin D levels constitute a risk factor for RRTIs, with serum 25(OH)D levels being significantly lower in children with recurrent infections compared to healthy controls [69]. Furthermore, hypocalcemia has also been identified as a risk factor associated with RRTIs [70]. Collectively, these studies indicate that dietary bias in preschool children may lead to nutritional deficiencies and inadequate intake of specific nutrients, ultimately increasing the risk of RRTIs. However, the underlying mechanisms require further investigation for validation.

The findings of this study suggest that daily outdoor activity time ≤ 2 h, co-residence with individuals having chronic respiratory disease, and preterm birth are not risk factors for recurrent respiratory tract infections in preschool children, which differs from the perspectives of some previous studies. Studies have found that higher physical activity levels in preschool children are significantly associated with fewer days of upper respiratory tract infection symptoms, while low physical activity levels may increase susceptibility to respiratory tract infections [71]. Considering the high heterogeneity associated with this factor, it can be inferred that the impact of outdoor activity on immunity may be characterized by "quality outweighing duration." A short duration of regular outdoor activity (e.g., one hour of outdoor play daily) may suffice to meet immune regulation requirements. Due to the high heterogeneity among the included studies, it was not possible to distinguish between the effects of "duration" and "quality," and the pooled results ultimately showed no significant association. Meanwhile, indoor activities with adequate ventilation may also reduce infection risk; therefore, insufficient duration per se may not constitute an independent risk factor. Studies have indicated that recurrent respiratory tract infections may be associated with chronic inflammation or abnormal immune responses [72]. If co-residing household members have chronic respiratory diseases, this may indirectly increase the risk of infection in children through genetic or environmental pathways. However, the present study showed that co-residence with individuals having chronic respiratory disease was not an independent risk factor. Possible explanations include the following: most chronic respiratory diseases are non-communicable, with extremely low pathogen shedding during stable phases; additionally, the compensatory effect of household protective behaviors may mitigate exposure risk. Therefore, future research still needs to further investigate the specific impact of the co-residence environment on recurrent respiratory tract infections in preschool children. Multiple studies have demonstrated that the incidence of lower respiratory tract infections in preterm infants at 1–2 years of age is significantly higher than that in term infants [73], and the proportion of moderate-to-late preterm infants hospitalized for lower respiratory tract infections is significantly higher than that of term infants [74]. Conversely, the results of this study indicated that preterm birth was not a risk factor for recurrent respiratory tract infections in preschool children. This may be attributed to substantial variability in follow-up durations among the included studies — for instance, some studies only followed up to infancy, while others extended into the preschool period — resulting in inconsistent temporal associations between exposure and outcome and consequently unstable pooled effect estimates. Meanwhile, given the high heterogeneity, it can be inferred that the impact of preterm birth on respiratory tract infections is age-dependent. Specifically, the risk may be higher during infancy, but as children enter the preschool period, with improved lung function and immune maturation, their infection risk gradually approaches that of term-born children. However, due to the substantial heterogeneity among the included studies, this dynamic change could not be precisely captured, and the pooled results ultimately showed no significant association.

### Implications for clinical practice

Based on the risk factors identified in this systematic review, we propose the following clinical management recommendations for RRTIs in preschool children for the consideration of frontline pediatricians: (1) Risk assessment and stratified management: Risk factors are categorized into non-modifiable factors (age, preterm birth/low birth weight, family history of allergy) and modifiable factors (vitamin A/D deficiency, zinc deficiency, secondhand smoke exposure, insufficient outdoor activity, unhealthy dietary patterns, and antibiotic overuse). It is recommended that priority be given to developing individualized intervention plans targeting the latter. (2) Nutritional support: children should receive adequate daily intake of vitamin D, zinc, and other essential trace elements. In addition, guidance on balanced dietary patterns should be provided to ensure sufficient consumption of vegetables, fruits, and high-quality protein, while limiting the intake of high-sugar processed foods. (3) Environmental and lifestyle interventions: Parents are strongly advised to quit smoking to ensure a smoke-free environment. At least 1–2 h of outdoor activity per day is recommended to enhance physical fitness and promote endogenous vitamin D synthesis. (4) Immunomodulation and prevention: Bacterial lysates (such as OM-85) have been shown to reduce the frequency and severity of RRTIs, with a relatively high level of evidence supporting their use [75]. (5) Antibiotic stewardship: Routine antibiotic use should be avoided for non-bacterial upper respiratory tract infections. Antibiotics should only be prescribed when bacterial infection is clearly confirmed or highly suspected, and should be administered in accordance with established guidelines. In summary, the management of RRTIs should follow the principle of "risk stratification→targeted intervention→dynamic follow-up" with modifiable factors as the primary focus of intervention, while integrating nutritional support, environmental measures, immunomodulation, and rational antibiotic use to reduce the risk of recurrent infections.

### Limitations

This study has several limitations that should be considered when interpreting the results. First, the exclusive inclusion of studies conducted in China represents one of the most important limitations of this systematic review. China's genetic background, environmental exposures, lifestyle patterns, healthcare system, and the epidemiological spectrum of respiratory tract pathogens differ substantially from those in other countries and regions, all of which may influence the risk factor profile for RRTIs and the magnitude of their effects. Therefore, the findings of this study should be interpreted as a summary of evidence based on the Chinese population, and extrapolation to other populations requires caution. Despite this geographical limitation, China is among the countries with the highest burden of childhood RRTIs globally, and conducting evidence synthesis based on the domestic population holds significant public health importance. A systematic review based on the Chinese population serves as an important complement to the international evidence base. Furthermore, given that the definition of RRTIs varies considerably across different countries and guidelines, the inclusion of multi-national studies would introduce greater definitional heterogeneity, potentially compromising the clinical interpretability of the pooled effect estimates. Based on the above analysis, we suggest that future research could explore the following directions: (1) Multi-country comparative studies: Conduct multi-center international collaborative studies using unified RRTIs definitions and study designs across different countries and regions, to clarify regional differences in risk factors and their underlying causes. (2) Establishment of standardized definitions: Promote the development of internationally unified diagnostic criteria for RRTIs, to establish a methodological foundation for cross-national comparative research. Second, all included studies were case-control studies, which are susceptible to recall bias (e.g., parental recall bias regarding breastfeeding duration and history of antibiotic use). In addition, the definitions of certain exposures were not uniform across studies (e.g., dietary bias and the definition of preterm birth), which may have contributed to increased heterogeneity. Furthermore, studies with positive results are more likely to be published, which may have overestimated the strength of association for some risk factors.

## Conclusion

Current evidence indicates that the occurrence of RRTIs in preschool children results from the interplay of multiple factors. Asthma, allergy, dietary bias, smoking co-resident, low parental education level, initial use of antibiotics < 6 months, maternal BMI per 3 kg/m² increment, and breastfeeding duration < 6 months are risk factors for recurrent respiratory tract infections in preschool children. In clinical and public health practice, priority should be given to implementing stratified interventions targeting identified risk factors, standardizing exposure assessment, and controlling for confounding factors. This approach will enable further validation of cross-factor interactions and associations within specific subgroups, thereby providing a more refined evidence-based basis for the clinical prevention and management of RRTIs.

## Authors’ contributions

Haizhu Huang: Conceptualization, Data curation, Formal Analysis, Investigation, Methodology, Project administration, Resources, Software, Validation, Visualization, Writing - original draft, Writing - review and editing. Xiaozhan Chen: Conceptualization, Data curation, Formal Analysis, Investigation, Methodology, Software, Writing - original draft, Writing - review and editing. Yi Wang: Data curation, Investigation, Resources, Validation, Visualization, Writing - review and editing. Dongxu Wang: Investigation, Resources, Supervision, Validation, Writing - review and editing. Chuanlin Zhou: Conceptualization, Funding acquisition, Methodology, Supervision, Writing - original draft, Writing - review and editing.

## Generative AI statement

Generative AI and AI-assisted technologies were NOT used in the preparation of this work.

## Funding

The author(s) declare that no financial support was received for the research, authorship, and/or publication of this article.

## Conflicts of interest

The authors declare that the research was conducted in the absence of any commercial or financial relationships that could be construed as a potential conflict of interest.

## Data Availability

The original contributions presented in the study are included in the article/Supplementary material, further inquiries can be directed to the corresponding author.

## Appendix Supplementary materials

Image, application 1
