# Supplementary material for: Prevalence and risk factors of recurrent respiratory tract infections in preschool children: a systematic review and meta-analysis
Source: J Pediatr (Rio J). 2026 Sep 5;102(6):101598. doi: 10.1016/j.jped.2026.101598 (PMC13571998; doi:10.1016/j.jped.2026.101598)
Supplement: Supplementary file 1 [file mmc1.docx]

**JPED-D-26-00105_Supplementary Material**

**Supplementary Tables**

**Table S1 Embase Retrieval strategy.**

| No | Query |
| --- | --- |
| #1 | ('preschool child'/exp OR 'preschool child') AND [embase]/lim |
| #2 | ('child, preschool'/exp OR 'child, preschool' OR 'pre-school child'/exp OR 'pre-school child' OR 'pre-school going children'/exp OR 'pre-school going children' OR 'pre-schooler'/exp OR 'pre-schooler' OR 'pre-schoolers'/exp OR 'pre-schoolers' OR 'preschool child institution'/exp OR 'preschool child institution' OR 'preschooler'/exp OR 'preschooler' OR 'preschool child'/exp OR 'preschool child') AND [embase]/lim |
| #3 | #1 OR #2 |
| #4 | ('recurrent respiratory tract infection'/exp OR 'recurrent respiratory tract infection') AND [embase]/lim |
| #5 | ('risk factor'/exp OR 'risk factor') AND [embase]/lim |
| #6 | ('relative risk'/exp OR 'relative risk' OR 'risk factors'/exp OR 'risk factors' OR 'risk factor'/exp OR 'risk factor') AND [embase]/lim |
| #7 | #5 OR #6 |
| #8 | #3 AND #4 AND #7 |

**Table S2 Web of science Retrieval strategy.**

| No | Query |
| --- | --- |
| #1 | (((((((TI=(Child, Preschool)) OR TI=(Preschool Child)) OR TI=(Children, Preschool)) OR TI=(Preschool Children)) OR AB=(Child, Preschool)) OR AB=(Preschool Child)) OR AB=(Children, Preschool)) OR AB=(Preschool Children) |
| #2 | (TI=(recurrent respiratory tract infection)) OR AB=(recurrent respiratory tract infection) |
| #3 | (((((((((((((((((((((((((((((((((((((TI=(Risk Factors)) OR TI=(Factor, Risk)) OR TI=(Risk Factor)) OR TI=(Population at Risk)) OR TI=(Populations at Risk)) OR TI=(Risk Scores)) OR TI=(Risk Score)) OR TI=(Score, Risk)) OR TI=(Risk Factor Scores)) OR TI=(Risk Factor Score)) OR TI=(Score, Risk Factor)) OR TI=(Health Correlates)) OR TI=(Correlates, Health)) OR TI=(Social Risk Factors)) OR TI=(Factor, Social Risk)) OR TI=(Factors, Social Risk)) OR TI=(Risk Factor, Social)) OR TI=(Risk Factors, Social)) OR TI=(Social Risk Factor)) OR AB=(Risk Factors)) OR AB=(Factor, Risk)) OR AB=(Risk Factor)) OR AB=(Population at Risk)) OR AB=(Populations at Risk)) OR AB=(Risk Scores)) OR AB=(Risk Score)) OR AB=(Score, Risk)) OR AB=(Risk Factor Scores)) OR AB=(Risk Factor Score)) OR AB=(Score, Risk Factor)) OR AB=(Health Correlates)) OR AB=(Correlates, Health)) OR AB=(Social Risk Factors)) OR AB=(Factor, Social Risk)) OR AB=(Factors, Social Risk)) OR AB=(Risk Factor, Social)) OR AB=(Risk Factors, Social)) OR AB=(Social Risk Factor) |
| #4 | #1 AND #2 AND #3 |

**Table S3 PubMed Retrieval strategy.**

| No | Query |
| --- | --- |
| #1 | "Child, Preschool"[Mesh] |
| #2 | ((Preschool Child) OR (Children, Preschool)) OR (Preschool Children) |
| #3 | ("Child, Preschool"[Mesh]) OR (((Preschool Child) OR (Children, Preschool)) OR (Preschool Children)) |
| #4 | recurrent respiratory tract infection |
| #5 | "Risk Factors"[Mesh] |
| #6 | (((((((((((((((((Factor, Risk) OR (Risk Factor)) OR (Population at Risk)) OR (Populations at Risk)) OR (Risk Scores)) OR (Risk Score)) OR (Score, Risk)) OR (Risk Factor Scores)) OR (Risk Factor Score)) OR (Score, Risk Factor)) OR (Health Correlates)) OR (Correlates, Health)) OR (Social Risk Factors)) OR (Factor, Social Risk)) OR (Factors, Social Risk)) OR (Risk Factor, Social)) OR (Risk Factors, Social)) OR (Social Risk Factor) |
| #7 | ("Risk Factors"[Mesh]) OR ((((((((((((((((((Factor, Risk) OR (Risk Factor)) OR (Population at Risk)) OR (Populations at Risk)) OR (Risk Scores)) OR (Risk Score)) OR (Score, Risk)) OR (Risk Factor Scores)) OR (Risk Factor Score)) OR (Score, Risk Factor)) OR (Health Correlates)) OR (Correlates, Health)) OR (Social Risk Factors)) OR (Factor, Social Risk)) OR (Factors, Social Risk)) OR (Risk Factor, Social)) OR (Risk Factors, Social)) OR (Social Risk Factor)) |
| #8 | ((("Child, Preschool"[Mesh]) OR (((Preschool Child) OR (Children, Preschool)) OR (Preschool Children))) AND (recurrent respiratory tract infection)) AND (("Risk Factors"[Mesh]) OR ((((((((((((((((((Factor, Risk) OR (Risk Factor)) OR (Population at Risk)) OR (Populations at Risk)) OR (Risk Scores)) OR (Risk Score)) OR (Score, Risk)) OR (Risk Factor Scores)) OR (Risk Factor Score)) OR (Score, Risk Factor)) OR (Health Correlates)) OR (Correlates, Health)) OR (Social Risk Factors)) OR (Factor, Social Risk)) OR (Factors, Social Risk)) OR (Risk Factor, Social)) OR (Risk Factors, Social)) OR (Social Risk Factor))) |

**Tables S4 The score of the Newcastle-Ottawa Scale quality assessment for included studies.**

| Author | Selection | Comparability | Outcome | Total NOS score |
| --- | --- | --- | --- | --- |
| Zhou B [16] | ☆☆☆ | ☆☆ | ☆☆☆ | 8 |
| Jin HX [17] | ☆☆ | ☆☆ | ☆☆☆ | 7 |
| Liu PH [18] | ☆☆☆ | ☆☆ | ☆☆☆ | 8 |
| Chen YN [19] | ☆☆☆ | ☆☆ | ☆☆☆ | 8 |
| Qi F [20] | ☆☆☆ | ☆☆ | ☆☆☆ | 8 |
| Tao SZ [21] | ☆☆ | ☆☆ | ☆☆☆ | 7 |
| Li XY [22] | ☆☆☆ | ☆☆ | ☆☆☆ | 8 |
| Zhou LM [23] | ☆☆☆ | ☆☆ | ☆☆☆ | 8 |
| He J [24] | ☆☆ | ☆☆ | ☆☆☆ | 7 |
| Li SF [25] | ☆☆ | ☆☆ | ☆☆☆ | 7 |

Tip: One star (☆) corresponds to 1 point.

**Supplementary Figure S1** **Funnel plots for assessment of publication bias.**

Funnel plots are presented for: (A) prevalence, (B) asthma, (C) dietary bias, (D) co-resident with chronic respiratory disease, (E) Preterm birth, and (F) daily outdoor activity time ≤2 hours. The symmetrical distribution of points within the pseudo 95% confidence limits (dashed lines) indicates a low risk of publication bias and supports the robustness of the meta-analysis results for all examined associations.

**Supplementary Figure S2 Sensitivity analysis using the leave-one-out method.**

Sensitivity analyses are shown for: (A) prevalence, (B) asthma, (C) dietary bias, (D) co-resident with chronic respiratory disease, (E) preterm birth, and (F) daily outdoor activity time ≤2 hours. Each plot displays the pooled estimate (dot) and 95% confidence interval (horizontal line) when individual studies were sequentially omitted. The stability of the overall estimates across all analyses confirms the robustness of the meta‐analysis results.
